# Supplementary material for: Tuning Penta-Graphene Electronic Properties Through Engineered Line Defects
Source: Sci Rep. 2020 May 15;10:8014. doi: 10.1038/s41598-020-64791-x (PMC7229116; doi:10.1038/s41598-020-64791-x)
Supplement: Supplementary file 1 — Supplementary information. [file 41598_2020_64791_MOESM1_ESM.pdf]

Electronic Supporting Information
For
"Tuning Penta-Graphene Electronic Properties Through Engineered Line Defects"

Ramiro Marcelo dos Santos[1], Leonardo Evaristo de Sousa[2], Douglas Soares Galvão[3], Luiz Antonio Ribeiro Junior[1]

[1]Institute of Physics, University of Brasilia, 70.919-970, Brasilia, Brazil

[2]Theoretical and Structural Chemistry Group, State University of Goias, 75133-050 Anapolis, Brazil

[3]Applied Physics Department, State University of Campinas, Campinas, SP, 13083-959, Brazil

(Dated: January 16, 2020)

Projected Density of States: sp^3 Doping Scheme

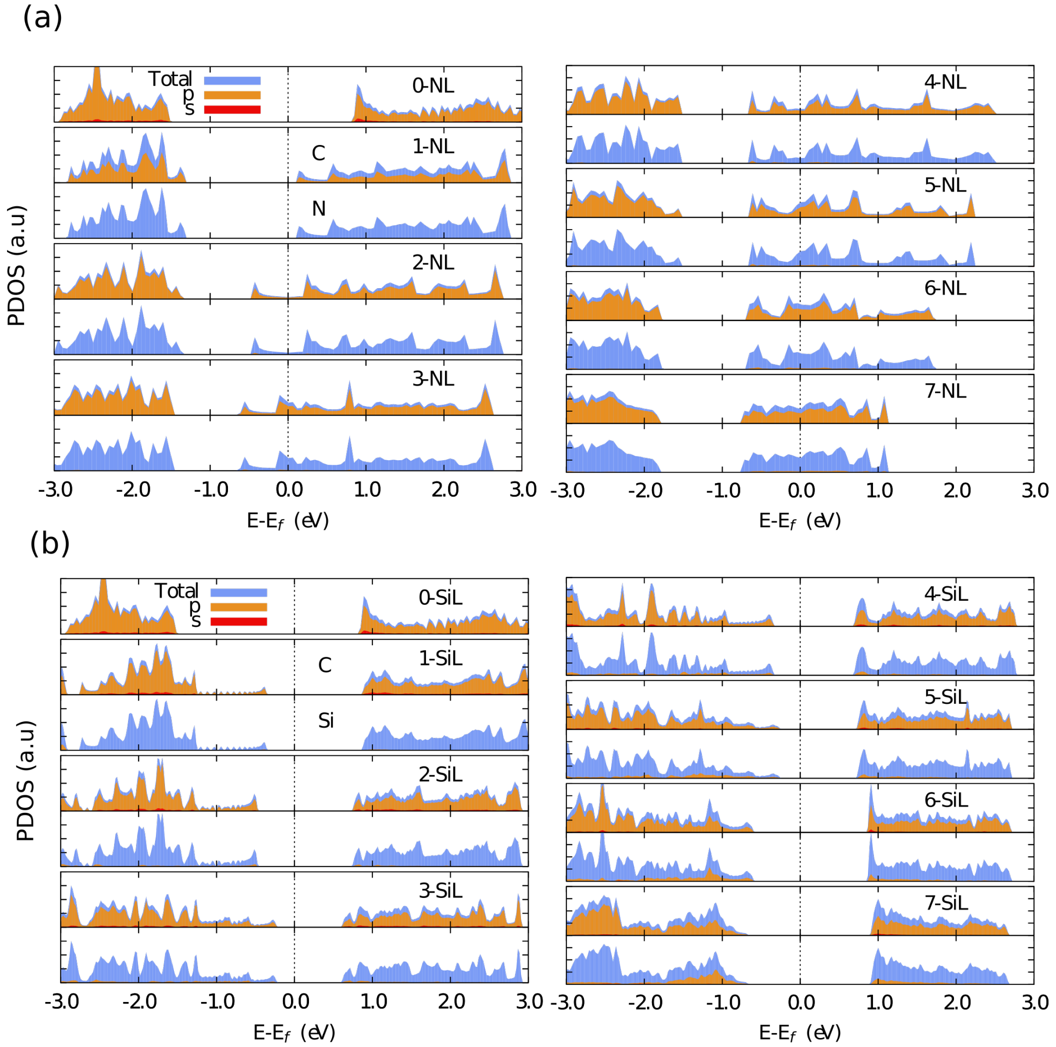

Figure1-ESI: Projected Density of States (PDOS) for all schemes of sp^3 carbon doping considering both (a) Nitrogen and (b) Silicon atoms. In this figure, X-NL and X-SiL denote the number (X) of dopant lines systemttcally inserted into the pentra-graphene structure.

Projected Density of States: sp^2 Doping Scheme

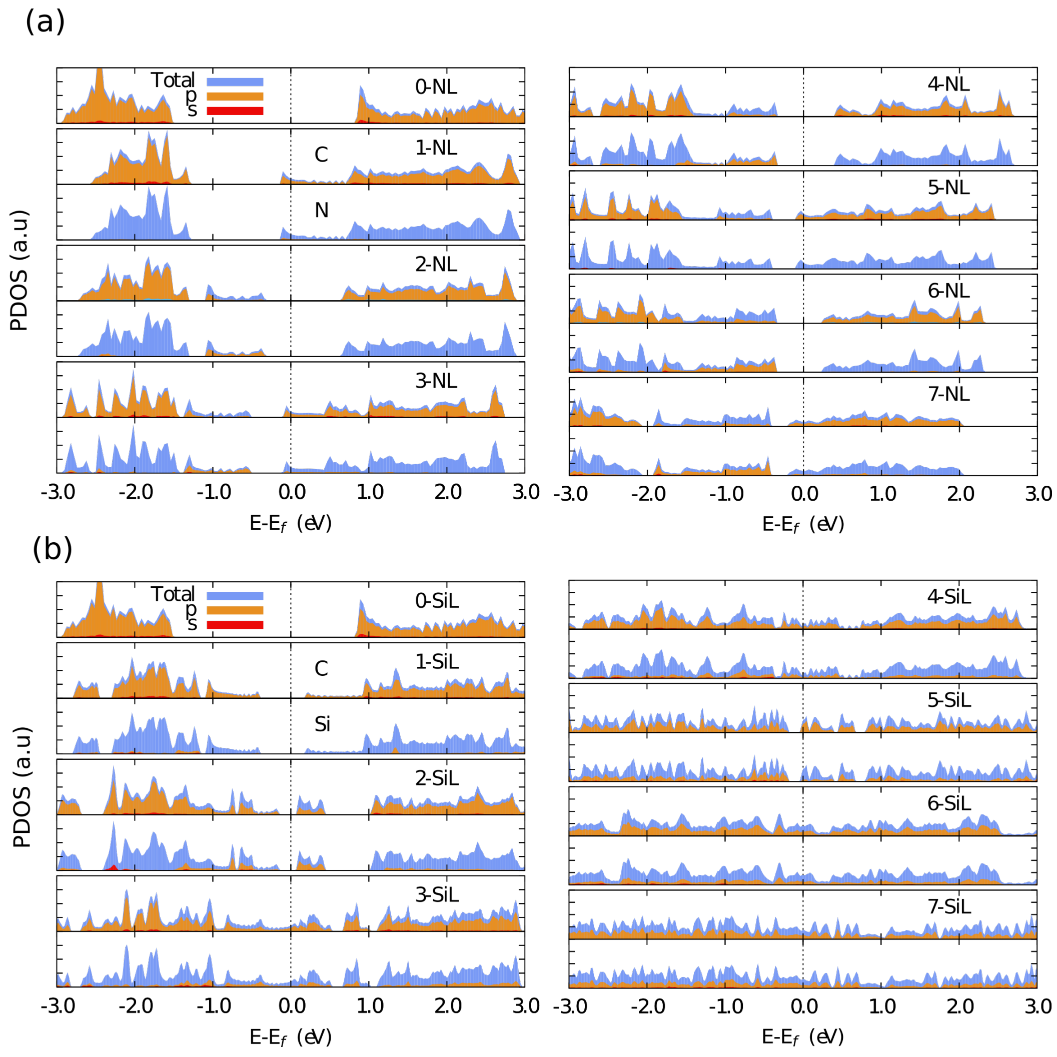

Figure2-ESI: Projected Density of States (PDOS) for all schemes of sp^2 carbon doping considering both (a) Nitrogen and (b) Silicon atoms. In this figure, X-NL and X-SiL denote the number (X) of dopant lines systemttcally inserted into the pentra-graphene structure.
